# Dr. Albert Nelson Chapman

**Published:** 1898-05

**Authors:** 


### Obituary.

### DR. ALBERT NELSON CHAPMAN.

The death of Dr. Albert Nelson Chapman occurred in Brooklyn, N. Y., on Wednesday, March 23, 1898. Although he had been ill for nearly three weeks with a severe attack of grip complicated with nerve trouble, no apprehension was felt of any fatal ending of his illness. He was considered convalescent, and on the day he died took a few steps around his room about 11.30 o'clock in the forenoon. Remonstrated with by his nurse for exposing himself, he consented to return to bed, remarking, "Yes, I want to rest," the last words which he ever spoke. In less than an hour, apparently asleep and lying quietly upon his side, he died from the bursting of a small blood-vessel in the brain.

Dr. Chapman was born in West Hampton, Mass., on March 27, 1838. He entered upon his professional work in Brooklyn in 1865, and for thirty-three years occupied the same suite of apartments in the Athenæum Building, corner of Clinton Street and Atlantic Avenue. He had strong artistic tastes, and his rooms were filled with choice pictures and works of art.

Professionally it was conceded that no one stood higher, his work being a synonyme for excellence. His practice was always extensive, and among the best families in the city. His patients, too, became, as a rule, his lifelong friends. His professional pride and conscientiousness was excessive.

During 1874–75 Dr. Chapman was President of the Second District Dental Association, and in 1877 was elected Librarian of the same organization.

Dr. Chapman's geniality of disposition and manner, his tender nature, and cheery helpful spirit greatly endeared him to every one who made his acquaintance. With a great wealth of floral offerings sent to his office from every direction, his remains were taken to his old home in West Hampton for interment on Saturday, March 26. On the day following he would have been sixty years of age. He leaves both in Massachusetts and in New York hosts of friends to remember him with the greatest tenderness, and to mourn him with sincerest sorrow.

C. B. L. R.

